# Risk Factors Associated with Increased Morbidity in Living Liver Donation

**DOI:** 10.1155/2015/949674

**Published:** 2015-12-15

**Authors:** Helry L. Candido, Eduardo A. da Fonseca, Flávia H. Feier, Renata Pugliese, Marcel A. Benavides, Enis D. Silva, Karina Gordon, Marcelo Gama de Abreu, Jaume Canet, Paulo Chapchap, Joao Seda Neto

**Affiliations:** ^1^Hepatology and Liver Transplantation, Hospital Sírio-Libanês, Rua Barata Ribeiro 414, cj 65, 01308-000 Bela Vista, SP, Brazil; ^2^Hepatology and Liver Transplantation, A.C. Camargo Cancer Center, São Paulo, SP, Brazil; ^3^Department of Anesthesiology, Hospital Sírio-Libanês, Rua Barata Ribeiro 414, cj 65, 01308-000 Bela Vista, SP, Brazil; ^4^Department of Anesthesiology, A.C. Camargo Cancer Center, São Paulo, Brazil; ^5^Department of Anesthesiology and Intensive Care Therapy, Pulmonary Engineering Group, University Hospital Carl Gustav Carus, Technische Universität Dresden, Dresden, Germany; ^6^Department of Anesthesiology, Hospital Universitari Germans Trias i Pujol, Badalona, Barcelona, Spain

## Abstract

Living donor liver donation (LDLD) is an alternative to cadaveric liver donation. We aimed at identifying risk factors and developing a score for prediction of postoperative complications (POCs) after LDLD in donors. This is a retrospective cohort study in 688 donors between June 1995 and February 2014 at Hospital Sírio-Libanês and A.C. Camargo Cancer Center, in São Paulo, Brazil. Primary outcome was POC graded ≥III according to the Clavien-Dindo classification. Left lateral segment (LLS), left lobe (LL), and right lobe resections (RL) were conducted in 492 (71.4%), 109 (15.8%), and 87 (12.6%) donors, respectively. In total, 43 (6.2%) developed POCs, which were more common after RL than LLS and LL (14/87 (16.1%) versus 23/492 (4.5%) and 6/109 (5.5%), resp., *p* < 0.001). Multivariate analysis showed that RL resection (OR: 2.81, 95% CI: 1.32 to 3.01; *p* = 0.008), smoking status (OR: 3.2, 95% CI: 1.35 to 7.56; *p* = 0.012), and blood transfusion (OR: 3.15, 95% CI: 1.45 to 6.84; *p* = 0.004) were independently associated with POCs. RL resection, intraoperative blood transfusion, and smoking were associated with increased risk for POCs in donors.

## 1. Introduction

In order to mitigate the shortage of cadaveric organs for liver transplantation, Raia et al. [1] and Broelsch et al. [2] introduced the techniques of reduced-size and split liver transplantation and resecting left lateral segments (LLS) from living adults for transplantation into children. Living donor liver transplantation (LDLT) was then introduced into clinical practice and subsequently expanded to adult patients after the first right lobe (RL) donation in 1990 [3].

The prerequisite to performing LDLT, however, is reduced morbidity and mortality risks of the donor [4]. Providing potential liver donors accurate and timely information regarding the risks associated with living donor liver donation (LDLD) is hampered by the lack of standardized reporting systems [5]. Additionally, under reporting of technical complications, blood and blood product transfusions and aborted donations all contribute to the lack of reliable information about the risks involved in LDLD [6].

In the present study, we aimed at identifying risk factors associated with postoperative complications (POCs) after LDLD in donors in two tertiary care center. We hypothesized that the risk of postoperative complications in this donor population is influenced by preexisting comorbidities, type of resection, and intraoperative characteristics.

## 2. Materials and Methods

The sample selection for this manuscript was based in the collected experience of 697 living donor liver resections performed at Sirio-Libanes Hospital and AC Camargo Cancer Center between June 1995 and February 2014. It allowed the assessment of 193 POCs, of which 43 were graded ≥III according to the Clavien-Dindo classification; 688 donors had records with complete data, which were retrospectively reviewed through patient charts and from a prospectively collected database. The hospital's ethics committee approved this study's protocol (HSL 2011-21).

The variables studied included the following: type of liver resection (LLS, LL, RL), living donor's age, gender, American Society of Anesthesiology (ASA) classification, presence of comorbidities, body mass index (BMI), and intraoperative packed red blood cell transfusion (PRBCT). The patients were evaluated for the development of POCs according to the Clavien-Dindo classification [7]. The primary outcome was the development of POCs ≥ grade III according to the Clavien-Dindo classification among the three types of liver resections performed. The secondary outcomes were as follows: intensive care unit (ICU) stay and hospital stay, reoperation, and readmission.

The preoperative, intraoperative, and postoperative protocols changed and evolved over the years. From the beginning of the experience until 2004, a Cell Saver (CellSaver) was routinely used during the live donor liver resections. During this period, autologous blood was collected a week before surgery to be used during the operation. These practices were used to ensure donor safety in the early stages of the group's experience with these procedures. After 2004, this protocol was abandoned and homologous PRBCT was used only when needed during the operation. In the outcome analysis, independently of the nature of blood transfusion, patients requiring blood were classified as PRBCT group.

### 2.1. Classification of Complications

Complications that occurred within 3 months from surgery were categorized according to the Clavien-Dindo classification for postoperative events [7]. Patients who developed more than one complication were graded according to the more severe type.

The POCs were divided into the following categories: bile leaks, being infectious (abdominal collection), being gastrointestinal (prolonged ileus, gastroparesis), liver necrosis, wound complications (wound infection, incisional hernia), deep venous thrombosis (DVT), being cardiovascular (atrial flutter, hypertension, endocarditis), pulmonary (pleural effusion, pneumothorax, bronchopneumonia, pulmonary thromboembolism), being hemorrhagic (intra-abdominal bleeding), and others.

### 2.2. Preoperative Donor Evaluation

The voluntary intent of the donor was first assessed and informed consent was mandatory. All donors underwent psychological evaluation to rule out any psychological disorders, coercion, or commercial motives. The preoperative evaluation of the candidates included routine blood tests, ABO system compatibility, urine analysis, electrocardiography, and chest X-ray. They were also tested for hepatitis A, B, and C serology, HIV, HTLV, CMV, EBV, syphilis, and Chagas disease. Abdominal Doppler ultrasound was performed to evaluate vascular anatomy, liver echogenicity (detection of steatosis and parenchymal lesions), and liver volumetry for LLS donation. For LL and RL resection, the anatomical and volumetric evaluation was performed with magnetic resonance imaging (MRI) and cholangio-MRI. The ratio of liver weight/recipient weight was used to estimate the preoperative graft-to-recipient weight ratio (GRWR). A GRWR > 1% was aimed when transplanting adolescents and adult recipients. When planning the donor operation, the accepted remaining liver volume calculated by the image studies was 30%.

Candidates with significant comorbidities, defined as ≥ASA III, were excluded as possible donors. Only donors younger than 50 years of age with a BMI less than 28 kg/m^2^ were accepted for surgery. All donors were preoperatively evaluated by an anesthesiologist and classified according to the ASA physical status classification [8]. Donors who were current smokers were encouraged to stop smoking 4 weeks before surgery, even though they were considered active smokers in this analysis.

### 2.3. Operative Techniques

The operative technique for the donor hepatectomy followed principles described elsewhere [9]. Parenchymal transection was accomplished with a Cavitron ultrasound surgical aspirator (Cavitron, Stanford, CA) after dissection of the hepatic hilum. The transection line on the liver varied with the graft type. Intraoperative cholecystectomy and cholangiography were performed to determine where to cut the donor's bile duct. Conventional clamping, cutting, and suturing of the major vessels were performed during graft removal. After the hepatectomy, the graft was flushed with Euro-Collins solution or histidine-tryptophan-ketoglutarate solution at 4°C and prepared for implantation.

### 2.4. Postoperative Management

All the donors recovered in the ICU during the immediate postoperative period. A Jackson-Pratt abdominal drain was routinely inserted at the end of the hepatectomy, close to the cut surface of the liver, to monitor for bleeding and bile leaks. The drain was removed only after day five if it showed a low output and serous aspect. The donors routinely received DVT prophylaxis with heparin 5000 UI every 12 hours, which was maintained until they were discharged from the hospital. The use of proton pump inhibitors continued for 30 days after surgery. If an ischemic residual liver segment was detected by clinical inspection at the end of the liver resection in cases of LLS, a 14-day course of ciprofloxacin was administered. This practice was introduced after 2007. Before that, the management of this occurrence included liver segment resection after completion of the hepatectomy, as previously reported [10].

### 2.5. Statistical Analysis

Means and medians were calculated to summarize continuous variables and were compared using *t*-tests or appropriate nonparametric tests when distributional assumptions were in doubt. Categorical variables were expressed as numbers and percentages. Differences between groups were assessed by chi-square or Fisher's exact tests, when needed.

Logistic regression was applied to the data and those variables found to be significant at *p* < 0.10 were selected for the multivariate modeling. Multivariate analysis was performed by stepwise forward logistic regression modeling. Variables were included in the model following a forward stepwise selection with a statistical critical level set at *p* < 0.05.

All analyses were performed using the SPSS 22.0 statistical package (IBM, Inc., Chicago, IL).

## 3. Results

**Figure 1:**
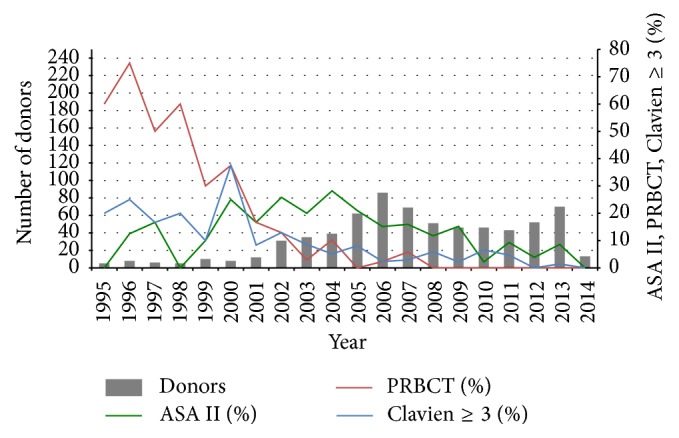
Distribution of donor operations, use of PRBCT, prevalence of ASA II donors, and prevalence of complications ≥ grade III of the Clavien-Dindo classification, from 1995 to 2013. ASA: American Society of Anesthesiology classification; PRBCT: packed red blood cell transfusion.

The number of LDLDs increased from 5 in 1995 to 69 in 2013, totaling 688 in the observation period (Figure 1). From these, 492 (71.4%) were LLS resections, 109 (15.8%) were LL, and 87 (12.6%) were RL. Overall, 346 (50.2%) of the donors were male, the mean BMI was of 23.6 ± 2.68 kg/m^2^, and 586 (86%) were classified as ASA I. Patients who were classified as ASA II (96 patients) presented the habits and comorbidities described in Table 1. Reasons for ASA II classification were not identified in 7 patients. Sixty-six (9.6%) donors received intraoperative PRBCT. The characteristics according to the type of liver resection are shown in Table 1.

When studying the donor's relationship with the recipient, it was observed that 223 (32.4%) were fathers, 240 (34.8%) were mothers, 58 (8.5%) were uncles/aunts, 50 (7.3%) were sons/daughters, 8 (1.2%) were grandfathers/grandmothers, 7 (1%) were husbands/wives, 60 (8.8%) were other relatives, and 39 (5.7%) were nonrelated.

A total of 193 out of 688 (28%) of the donors developed at least one POC; of the 193, 45 (23.3%) developed grade I, 105 (54.4%) developed grade II, 5 (2.5%) developed grade IIIa, 33 (17%) developed grade IIIb, and 5 (2.5%) developed grade IVa. Forty-three donors (6.2%) developed at least one POC ≥ grade III. Twenty-three patients (4.5%) in the LLS group, 6 (5.5%) patients in the LL group, and 14 (16.1%) patients in the RL group developed POCs ≥ grade III (*p* < 0.001).

A total of 242 POCs were documented in 193 patients. Most of the complications (52.8%) were grade II. The most frequently observed complication was bile leak (23.1%), but the majority (71.4%) was resolved without need of further intervention. The most serious complications reported were grade IVa, which occurred in 5 patients and included pulmonary thromboembolism, deep venous thrombosis, endocarditis, atrial flutter, and rhabdomyolysis. One patient presented portal vein thrombosis 6 months following RL donation, whereas 2 LLS donors committed suicide 5 years after the surgery. The POCs are shown in Table 2.

As shown in Table 3, bile leaks, wound complications, and pulmonary complications were more frequent with RL than other liver resection types. Also, the occurrence of at least one complication was more frequent in donors who underwent RL liver resection (23.9% LLS versus 33.9% LL versus 43.6% RL; *p* < 0.001) (Table 4). Donors submitted to LL and RL resections had longer ICU and hospital stays as well as readmission and reoperation rates when compared to donors who underwent LLS resection (Table 5).

Donors who smoked were more likely to develop POCs than nonsmokers (44.9% versus 26.7%; *p* = 0.007). Also, the frequency of complications ≥ grade III was higher in smokers (16.3% versus 5.5%; *p* = 0.008). The most frequently observed complication in this subgroup was bile leak, followed by liver necrosis and pulmonary and gastrointestinal complications (Table 6).

The variables initially included in the multivariate model were as follows: type of liver resection, gender, age, PRBCT, ASA classification, and smoking status. RL donation (OR: 2.81, 95% CI: 1.32 to 3.01; *p* = 0.008), smoking status (OR: 3.2, 95% CI: 1.35 to 7.56; *p* = 0.012), and PRBCT (OR: 3.15, 95% CI: 1.45 to 6.84; *p* = 0.004) were retained in the model (Table 7).

Figure 1 shows the prevalence of ASA II patients, the use of PRBCT, and the incidence of POCs ≥ grade III. In more recent years, the need for PRBCT has become minimal (1.8%) and the incidence of complications ≥ grade III has also dropped from 20–30% in the early years to 1.5–5% in recent years.

## 4. Discussion

The main findings of this retrospective analysis in 688 donors who underwent liver resection for LDLT were as follows: (1) the risk of severe POCs is higher in patients submitted to RL donation; (2) donors who were active smokers and those that received blood transfusion during surgery also have higher risk of developing severe POCs; (3) the most frequently observed complication was bile leaks.

Since the introduction of LDLT into clinical practice, the focus of the medical community has been to define the risk factors and establish procedures to ensure donor safety during the process of living donation. It is well known that there is a significant difference between the donor risks for RL/extended right donation and LL/LLS donation and that RL donors have more risk factors than non-RL donors [11]. Indeed, despite the improvements in the donor evaluation process for the careful screening of candidates for RL donation and more refinements in surgical techniques during the learning curve [12, 13], there has been no significant decrease in the rate of donor complications in recent years [12, 14, 15]. However, morbidity after LDLD strongly correlates with medical center experience and Broering et al. [13] have shown a significant decrease over time in perioperative morbidity from 53.8% to 9.2%, in a prior report. In practice, complete prevention of donor complications is not feasible [16].

In this report, RL donation was associated with an increased incidence of POCs when compared to LL or LLS. It was also independently associated with the occurrence of POCs ≥ grade III in the Clavien-Dindo classification. Iida et al. [11] recently reported their 17-year findings from over 1000 liver resections for LDLT (500 right grafts and 762 left liver grafts). The incidence and severity of complications were higher in cases of right grafts (44.2% versus 18.8%, *p* < 0.05). Donor age, RL donation, and prolonged operation time were found to be independent risk factors for complications following the operation. Interestingly, a recent report from the Japanese Liver Transplantation Society showed a much lower incidence of POCs after live liver donation [17]. The incidence of POCs in LL and RL donors was 8.7% and 9.4%, respectively, but grade I complications in the Clavien-Dindo classification were excluded. Nevertheless, it seems that the collective experience showed a reduction in the incidence of donor complications, especially for RL resection. Another limitation of the Japanese report is related to the lack of a complete reporting system, especially for Clavien-Dindo grade II and III complications, and thus donor morbidity may have been further underestimated. Effectively, a systematic review reported by Middleton et al. [18] showed that donor morbidity ranged from 0% to 100%, with a median of 16.1%. Ghobrial et al. [6], in the 9-center Adult-to-Adult Living Donor Liver Transplantation Cohort Study (A2ALL), reported a 38% rate of complications following RL donation. Biliary leaks beyond postoperative day 7 occurred in 36 (9%) patients, three patients experienced vascular problems (2 portal vein thrombosis, 1 inferior vena cava thrombosis), and 1 patient died due to complications related to the procedure.

The safety of a right hepatectomy (RH) with preservation of approximately one-third of the total normal liver volume [19] has been the main justification for the acceptance of this procedure for LDLT. Belghiti et al. [20] prospectively studied 32 patients requiring an RH for benign liver lesions (BL) matched with 32 living donors. They found that RH in LDLT led to a more severe deprivation of liver volume than in BL, which induced accelerated liver regeneration. The overall complication rate was 46% in the living donor group* versus* 21% in the BL group (*p* = 0.035). The authors argue that this accelerated regeneration could represent an “inherent limitation” in healthy donors that makes them more vulnerable to POCs. Similarly, Facciuto et al. [21], in a retrospective review of 137 RH for LDLT, showed a 42% complication rate in donors with a remnant liver volume <30% versus 31% in >30%.

In this report, the donor risk for developing Clavien-Dindo complications ≥ grade III increased 3.15 times when PRBCT was used intraoperatively. Similarly, Ghobrial et al. [6] showed that intraoperative blood transfusion was the only variable associated with a significantly higher risk of at least one complication among live donors in the 9-center A2ALL. Compared with donors who required no blood transfusions, those who received up to one unit were 2.7 times more susceptible to complications. A more recent publication from the A2ALL [22] in a cohort of 740 LDLD corroborated the results from the previous report: there was a significant association of transfusion requirements with the development of a first complication of any type (HR = 1.38 per unit; *p* < 0.0001) and specifically with the occurrence of bile leak and infection. Indeed, bile leaks, wound complications, and pulmonary complications were more frequent in RL liver resections in the present report. Lo [23] showed a series of 1508 cases of living liver donors where the rate of complications was 15.8%. In these cases, pulmonary complications were uncommon. Conversely, in smaller cohort of live donation, Dondero et al. [24] reported fourteen major respiratory complications in 11 of 112 donors (9.8%). In that study, pulmonary complications were frequent in living liver donors after the operation and pulmonary embolism was the most frequent of these complications. Of note, pulmonary morbidity was observed mainly following RH.

Ideally, candidates for living donation should be classified as ASA I [8]. In practice, most of the transplant centers allow ASA II candidates to proceed and go through with the operation because these patients present no functional limitations. In our cohort, 55% of the donors classified as ASA II were active smokers. Indeed, ASA II was found to be a surrogate marker of the effects of smoking. Donors who smoke have a risk 3.2 times higher than nonsmokers of developing POCs ≥ grade III, according to our findings. This is similar to the risk presented by the need for intraoperative PRBCT. The effects of smoking on cardiovascular and pulmonary systems as well as the detrimental effects on wound healing have already been described. Smokers have increased mucus production and the damage to tracheal cilia impairs the clearance of such mucus [25]. The impairment of the wound healing process can also be responsible for the higher risk of anastomotic leaks after colorectal surgery, presented by patients who smoke [26]. In our series, the most frequent complication in smokers was bile leaks and liver necrosis, followed by pulmonary complications. In fact, the distribution of types of complications was similar to those observed in the entire cohort. The effects of preoperative smoking cessation are controversial, as is the period necessary for this intervention to be beneficial. A decrease in wound complications was demonstrated in nonsmoking patients [27], but most of the studies show no difference in this outcome with smoking cessation [25]. In a large review [25] of studies of smoking cessation intervention prior to surgery, the incidence of cardiopulmonary complications in nonsmoking patients compared to current smokers did not differ significantly. It has been determined that pulmonary function recovers in 8 weeks after smoking cessation, but the minimal timing needed to decrease the risk of POCs has not been determined yet [25]. Nakagawa et al. [28] found that both current smokers and former smokers that stopped 4 weeks before pulmonary surgery had a higher risk of postoperative pulmonary complications than those who were never smokers. Our current practice with donors who smoke is to encourage a 4-week period of abstinence from smoking before surgery. The findings in the present paper raise the question if we should continue to accept smokers as donors, further prioritizing donor safety. Whether advising donors to abstain from smoking for a longer period before surgery would be enough to minimize the risk of complications remains to be determined. Interestingly, the presence of a smoking habit has not been reported in previous studies regarding the evaluation of complications following LDLD.

*Limitations*. This study has different limitations. First, this was a retrospective study, and donors were assessed by the same medical team. Results are likely affected by local characteristics, including the experience of the staff. Second, change in clinical practice during the observation period may have occurred, a fact that would influence the results.

## 5. Conclusions

POCs were relatively common in this cohort of LDLDs. RL donation, use of intraoperative PRBCT, and smoking were independently associated with the occurrence of POCs ≥ grade III in the Clavien-Dindo classification after live donation.

## Figures and Tables

**Table 1:** Preoperative and intraoperative variables according to the type of liver resection.

Variables	All	LLS	LL	RL	*p*
	*N* = 688	*N* = 492	*N* = 109	*N* = 87	

Age, mean ± SD	29.8 ± 7.20	28.7 ± 6.7	33.6 ± 8.2	31.2 ± 8.5	<0.001
Sex, male, number (%)	346 (50.3)	225 (45.7)	67 (61.5)	55 (63.2)	<0.001
BMI, mean ± SD	23.6 ± 2.7	23.4 ± 2.7	24 ± 2.3	23.9 ± 2.7	0.13

	*N* = 682	*N* = 492	*N* = 106	*N* = 84	

ASA, number (%)					0.36
I	586 (85.9)	427 (86.8)	91 (85.8)	68 (80.9)	
II	96 (14.1)	65 (13.2)	15 (14.2)	16 (19.1)	

	*N* = 89	*N* = 59	*N* = 15	*N* = 15	0.32

Smoker, number (%)	49 (55.0)	32 (54.1)	8 (53.3)	9 (60.0)	
Anemia, number (%)	7 (7.9)	6 (10.2)	1 (6.7)	0	
Asthma, number (%)	6 (6.7)	3 (5.1)	1 (6.7)	2 (13.3)	
Renal lithiasis, number (%)	4 (4.5)	4 (6.8)	0	0	
Overweight, number (%)	4 (4.5)	3 (5.1)	1 (6.7)	0	
MMVD, number (%)	2 (2.3)	2 (3.4)	0	0	
Hypertension, number (%)	4 (4.5)	1 (1.7)	2 (13.3)	1 (6.7)	
Gastritis, number (%)	2 (2.2)	0	0	2 (13.3)	
Other, number (%)^*∗*^	11 (12.4)	8 (13.6)	2 (13.3)	1 (6.7)	

	*N* = 688	*N* = 492	*N* = 109	*N* = 87	

PRBCT, number (%)	66 (9.6)	29 (5.9)	13 (11.9)	24 (27.6)	<0.001
Graft weight (g), mean ± SD		288.6 ± 60.9	433.8 ± 100.0	814.2 ± 269.8	<0.001

	*N* = 608	*N* = 432	*N* = 99	*N* = 77	

Number of bile ducts, number (%)					<0.001
1	479 (78.7)	354 (82.0)	82 (82.8)	43 (55.8)	
2	122 (20.1)	74 (17.1)	16 (16.2)	32 (41.6)	
3	7 (1.2)	4 (0.9)	1 (1.0)	2 (2.6)	

LLS, left lateral segment; LL, left lobe; RL, right lobe; BMI, body mass index; ASA, American Society of Anesthesiology physical status classification; PRBCT, packed red blood cell transfusion; MMVD, minor mitral valve dysfunction; SD, standard deviation.

^*∗*^hypothyroidism, hiatal hernia, dyslipidemia, upper airway disturbances, mood disorders, leucopenia, and arrhythmia.

**Table 2:** Classification distribution of postoperative complications according to Clavien-Dindo classification.

Grade	Bile leak	Infectious	GI	Liver necrosis	Wound	DVT	Cardio	Pulm	Bleeding	Other	Total, *n* (%)
I	5	12	17	0	11	0	0	3	0	1	49 (20.2)
II	35	24	19	8	5	2	2	10	0	23	128 (52.9)
IIIa	1	0	0	0	1	0	0	4	0	0	6 (2.5)
IIIb	15	10	4	13	5	1	0	4	1	1	54 (22.3)
IVa	0	0	0	0	0	1	2	1	0	1	5 (2.1)
Total, *n* (%)	56 (23.1)	46 (19.0)	40 (16.5)	21 (8.7)	22 (9.1)	4 (1.7)	4 (1.7)	22 (9.1)	1 (0.4)	26 (10.7)	242

Infectious: infectious complications; DVT: deep venous thrombosis; GI: gastrointestinal complications; Cardio: cardiac complications; Pulm: pulmonary complications. Some donors experienced two or more complications.

**Table 3:** Complication occurrence according to the type of liver resection.

Complication^*∗*^	LLS *N* = 492, no. (%)	LL *N* = 109, no. (%)	RL *N* = 87, no. (%)	*p*
Bile leak	33 (6.7)	10 (9.2)	13 (14.9)	0.01
Infectious	30 (6.1)	9 (8.3)	7 (8.0)	0.37
Liver necrosis	17 (3.5)	1 (0.9)	3 (3.4)	0.90
Wound	12 (2.4)	2 (1.8)	8 (9.2)	0.006
DVT	2 (0.4)	1 (0.9)	1 (1.1)	0.61
Gastrointestinal	24 (4.9)	10 (9.2)	6 (6.9)	0.04
Cardiologic	3 (0.6)	0	1 (1.1)	0.50
Pulmonary	10 (2.0)	3 (2.8)	9 (10.3)	<0.001
Others	12 (2.4)	8 (7.3)	6 (6.9)	0.01

^*∗*^in a total of 242 complications; LLS, left lateral segment; LL, left lobe; RL, right lobe; DVT, deep venous thrombosis. Some donors experienced two or more complications.

**Table 4:** Classification of complications according to Clavien-Dindo among the types of liver resection.

Complication grade	LLS *N* = 492, no. (%)	LL *N* = 109, no. (%)	RL *N* = 87, no. (%)
I	35 (7.1)	7 (6.4)	3 (3.4)
II	60 (12.2)	24 (22.0)	21 (24.1)
IIIa	1 (0.2)	2 (1.8)	2 (2.3)
IIIb	19 (3.9)	4 (3.7)	10 (11.5)
IVa	3 (0.6)	0	2 (2.3)
Total^*∗*^	118 (24.0)	37 (33.9)	38 (43.7)

^*∗*^in a total of 193 patients; LLS, left lateral segment; LL, left lobe; RL, right lobe.

If a donor experienced two or more complications, only the highest grade was recorded.

**Table 5:** Outcomes according to the type of liver resection.

Variables	LLS	LL	RL	*p*
	*N* = 492	*N* = 109	*N* = 87	

ICU (days), mean ± SD	1.1 ± 0.3	1.3 ± 0.7	1.7 ± 1.0	<0.001
Hospital stay (days), mean ± SD	6.1 ± 1.6	6.4 ± 1.7	7.2 ± 2.9	<0.001
Reoperation, number (%)	10 (2.0)	4 (3.7)	7 (8.0)	0.012

	*N* = 491	*N* = 107	*N* = 85	

Readmission, number (%)	30 (6.1)	8 (7.5)	13 (15.3)	0.018

LLS, left lateral segment; LL, left lobe; RL, right lobe.

**Table 6:** Classification of postoperative complications in smokers versus nonsmokers.

Complication	Smokers *n* = 49		Nonsmoker *n* = 639	
	Grades I + II number (%)	≥Grade III number (%)	Grades I + II number (%)	≥Grade III number (%)
Bile leak	9 (52.9)	3 (20.0)	31 (19.4)	13 (26.0)
Infectious	1 (5.9)	1 (6.7)	35 (21.9)	9 (18.0)
Liver necrosis	2 (11.8)	3 (20.0)	6 (3.8)	10 (20.0)
Wound	0	1 (6.7)	16 (10.0)	5 (10.0)
DVT	0	2 (13.3)	2 (1.3)	0
Gastrointestinal	2 (11.8)	1 (6.7)	34 (21.3)	3 (6.0)
Cardiologic	0	2 (13.3)	2 (1.3)	0
Pulmonary	1 (5.9)	2 (13.3)	12 (7.5)	7 (14.0)
Other	2 (11.8)	0	22 (13.8)	3 (6.0)
Total^*∗*^, *N*	17 (34.7)	15 (30.6)	160 (25)	50 (7.8)

^*∗*^in a total of 242 complications; DVT: deep venous thrombosis.

**Table 7:** Multivariate logistic regression analysis for the development of complications ≥ grade III.

Variables	*p*	OR	95% CI
Graft type			
LLS	—	—	—
LL	0.94	1.04	0.41 to 2.67
RL	0.008	2.81	1.32 to 6.01
Smoker (no)	—	1	—
Smoker (yes)	0.012	3.20	1.35 to 7.56
PRBCT (no)	—	1	—
PRBCT (yes)	0.004	3.15	1.45 to 6.84

LLS, left lateral segment; RL, right lobe; PRBCT, packed red blood cell transfusion; OR, odds ratio; CI, confidence interval.

## Abbreviations

LLS: Left lateral segment (liver segments II and III)
LDLT: Living donor liver transplantation
LDLD: Living donor liver donation
ASA: American Society of Anesthesiology
BMI: Body mass index
POC: Postoperative complication
PRBCT: Packed red blood cells transfusion
ICU: Intensive care unit
DVT: Deep venous thrombosis
MRI: Magnetic resonance imaging
GRWR: Graft-to-recipient weight ratio
LL: Left lobe (liver segments II, III, and IV)
LT: Liver transplant
RL: Right lobe (liver segments V, VI, VII, and VIII)
RH: Right hepatectomy.
